# Non-invasive detection and complementary diagnosis of liver metastases via chemokine receptor 4 imaging

**DOI:** 10.1038/s41417-022-00433-w

**Published:** 2022-02-10

**Authors:** Hua Yang, Shanshan Tan, Jingjuan Qiao, Yiting Xu, Zongxiang Gui, Yuguang Meng, Bin Dong, Guangda Peng, Oluwatosin Y. Ibhagui, Weiping Qian, Jimmy Lu, Zezhong Li, Guimin Wang, Jinping Lai, Lily Yang, Hans E. Grossniklaus, Jenny J. Yang

**Affiliations:** 1 Department of Ophthalmology, Emory University, Atlanta, GA 30322 USA; 2 Department of Chemistry, Georgia State University, Atlanta, GA 30303 USA; 3 Yerkes National Primate Research Center, Atlanta, GA 30329 USA; 4 Department of Biology, Georgia State University, Atlanta, GA 30303 USA; 5 Department of Surgery, Emory University, Atlanta, GA 30322 USA; 6 Codex BioSolutions Inc, Gaithersburg, MD USA; 7 Affiliated Eye Hospital of Shandong Traditional Chinese Medicine University, Jinan, China; 8 Department of Pathology and Laboratory Medicine, Kaiser Permanente Sacramento Medical Center, Sacramento, CA 95825 USA

**Keywords:** Biomarkers, Metastasis

## Abstract

Noninvasive detection of early-stage liver metastases from different primary cancers is a pressing unmet medical need. The lack of both molecular biomarkers and the sensitive imaging methodology makes the detection challenging. In this study, we observed the elevated expression of chemokine receptor 4 (CXCR4) in uveal melanoma (UM) patient liver tissues, and high CXCR4 expression in liver metastases of UM murine models, regardless of the expression levels in the primary tumors. Based on these findings, we identified CXCR4 as an imaging biomarker and exploited a CXCR4-targeted MRI contrast agent ProCA32.CXCR4 for molecular MRI imaging. ProCA32.CXCR4 has strong CXCR4 binding affinity, high metal selectivity, and r1 and r2 relaxivities, which enables the sensitive detection of liver micrometastases. The MRI imaging capacity for detecting liver metastases was demonstrated in three UM models and one ovarian cancer model. The imaging results were validated by histological and immunohistochemical analysis. ProCA32.CXCR4 has strong potential clinical application for non-invasive diagnosis of liver metastases.

## Introduction

Tumor metastasis causes about 90% of cancer related-deaths [1]. Cancer diagnosis, staging, and treatment stratification depend substantially on identifying the metastatic spread of primary tumors. The liver is a common site of metastases for different types of primary malignancies including uveal melanoma (UM), ovarian cancer, pancreatic cancer, and colon cancer [2, 3]. In a study of metastatic progression across 16 major cancer types, 59% of the cases demonstrated liver metastases [2], thereby highlighting the importance of developing non-invasive methods of early detection of liver metastases for cancer management.

UM almost exclusively metastasizes to the liver [4]. The progression of metastases in UM patients does not terminate even after surgical removal of the primary tumor [5]. Liver metastases can form before the removal of the primary cancer, remain dormant for years, and recur decades later [6]. Similar high recurrences are also found in liver metastases from pancreatic cancer, colon cancer, and ovarian cancer [7–9]. Liver metastases from UM are not responsive to immune checkpoint inhibitors despite recent successes with cutaneous melanoma [10]. Encouraging results have been reported with partial hepatectomy for early-stage solitary metastases [11]. A significant barrier to treatment is the lack of non-invasive staging methods for early-stage metastases, which is essential to understanding the biology of hepatic metastases, monitoring prognosis, and planning for personalized treatment.

Currently, there is no reliable way to detect liver metastases at a sufficiently early stage to improve survival. While PET is highly sensitive [12], its radioactivity, non-specific uptake at liver, and limited spatial resolution, are problematic for detecting small liver metastases. Biopsies are often not recommended to confirm the diagnosis of small lesions due to sampling errors, feasibility, targeting difficulty, and other complications [13]. Contrast-enhanced magnetic resonance imaging (MRI) and multidetector computed tomography (MDCT) are commonly used for detection of liver metastatic lesions. However, these methods cannot detect liver metastasis < 1 cm with high sensitivity and specificity [12, 14]. Despite significant effort in genetic profiling of tumors to reveal molecular markers that predict metastatic risk, currently there is no validated molecular biomarker that can be used for accurate imaging [10, 15–19]. An in vitro immunoreactivity assay based on the BRCA1-associated protein 1 (BAP1) mutation [19] has been suggested as a parameter to evaluate metastatic risk [18]. However, like other prognostic parameters such as chromosomal aberrations, BAP1 can only predict the potential metastases. Therefore, monitoring the development of liver metastases with high sensitivity and accuracy is an unmet medical need.

C-X-C chemokine receptor 4 (CXCR4) expression on tumor cells has been associated with an unfavorable progression and metastasis in cancers including melanoma, ovarian cancer, breast cancer, pancreatic cancer, and prostate cancer [17]. CXCR4 has been shown to play a crucial role in the dissemination and extraversion of various types of cancer cells and the formation of liver metastases. One explanation of this phenomenon is the high level of CXCL12 (the natural ligand of CXCR4) produced by hepatic sinusoidal endothelial cells and hepatic stellate cells in the liver. The CXCL12/CXCR4 interaction promotes the early event of primary cancer cells expressing CXCR4 migrating towards CXCL12 to the liver [17]. CXCR4 is also a therapeutic target for the FDA approved antagonist, Plerixafor, that has been suggested as a complementary treatment for ovarian cancer [20]. However, the expression of CXCR4 levels in primary cancers and metastases are inconsistent from different studies due to the lack of systematic investigation and quantification [21]. It is important to develop a non-invasive complementary diagnostic test for CXCR4-related therapeutics.

Here, we first report our observation of elevated CXCR4 expression in liver metastases in both human patients and animal models and identify CXCR4 is a valid imaging biomarker. We then report our development of a CXCR4 targeted protein contrast agent ProCA32.CXCR4, which exhibits significantly improved dual relaxivity and Gd^3+^ binding, compared to clinically approved contrast agents. We have achieved early detection of liver metastases originating from parental UM cells with differential expression of CXCR4, as well as primary ovarian cancer, with our CXCR4 targeted MRI contrast agent using metastatic murine models. Our discovery of the upregulation of CXCR4 expression in the hepatic metastases, and the novel imaging methodology are expected to fill the major gap in non-invasive and precise detection of early-stage liver metastases for cancer management.

## Materials and methods

### Cell lines and cell culture

All cell lines were cultured at 37 °C, 5% CO_2_ in a humidified incubator according to the standard protocol of mammalian cell culturing. Human UM cells 92.1, OCM1, OMM2.3, OMM3, M20-07-070, M20-09-196, Mel290, and Mel270, authenticated by STR (Emory Genomics core facility) [22] were cultured in RPMI 1640 with L-glutamine (Corning Cellgro, Albany, NY) supplemented with 10% fetal bovine serum (FBS) (Sigma-Aldrich, St. Louis, MO), Sodium Pyruvate (Cellgro, Albany, NY), MEM Non-Essential Amino Acids (Cellgro, Albany, NY), MEM Vitamins (Cellgro, Albany, NY), Penicillin-Streptomycin Solution (Cellgro, Albany, NY), and HEPES buffer (Corning, Albany, NY). The 92.1 and OMM2.3 cells were provided by Dr. Jerry Niederkorn (Department of Ophthalmology, UT Southwestern, Dallas, TX). The Mel290 and Mel270 cells were provided by Dr. Bruce Ksander (Schepens Eye Institute, Boston, MA). The OCM1 and OMM3 cells were donated by Dr. June Kan-Mitchel (Wayne State University, Detroit, MI). Dr. Scott Woodman (Department of Melanoma Medical Oncology and Systems Biology, MD Anderson Cancer Center, Houston, TX), Dr. Barry Burgess, and Dr. Tara McCannel (UCLA, Jules Stein Eye Institute, Calabasas, CA) isolated and provided M20-09-196 and M20-07-070 cell lines. SKOV3 ovarian cancer cells were cultured in McCoy 5 A medium with 10% fetal bovine serum and 1% penicillin and streptomycin.

### Flow cytometry analysis

Flow cytometry was used to analyze the CXCR4 expression of UM cell lines. UM cell lines were cultured until confluency reached 80–90%, then dissociated with non-enzymatic cell dissociation solution (Sigma-Aldrich, St. Louis, MO), washed three times with phosphate-buffered saline (PBS), and blocked with Human BD Fc Block™ (BD Biosciences, San Jose, CA) for 10 min at room temperature. Following blocking, UM cells were immunolabeled with recombinant anti-CXCR4 antibody [UMB2] (Catalog number: ab124824; Abcam, Cambridge, MA) for 20 min. Each experiment was performed triplicate. BD LSRFortessa™ Cell Analyzer (BD Biosciences, San Jose, CA) was used to perform flow analysis. FlowJo software (Tree Star, Ashland, OR) was used for data analysis.

### Immunohistochemical analysis

Single-labeling immunohistochemistry was performed using Autostainer (Dako, Carpentaria, CA) with the chain polymer-conjugated technology. After deparaffinization in 3 changes of xylene, rehydration in 100–80% alcohol, and antigen retrieval in 0.01 M sodium citrate buffer (pH 6.0) at 99–100 °C, tissue sections were blocked by Trident Universal Protein Blocking Reagent and incubated with recombinant anti-CXCR4 antibody [UMB2] (Catalog number: ab124824; Abcam, Cambridge, MA) at the dilution ratio of 1:250 and ready-to-use HMB45 (Catalog number: GA05261-2; Dako, Carpentaria, CA) and gp100 antibody (Catalog number: ab137078; Abcam, Cambridge, MA) at the dilution ratio of 1:100, and ready-to-use CK7 (Catalog number: IS61930-2; Dako, Carpentaria, CA), overnight at room temperature, followed by introduction of the labeled polymer (Dako, Carpentaria, CA), according to the manufacturer’s instructions. The red, brown or teal chromogen substrate was applied with hematoxylin as the counterstaining. A NanoZoomer 2.0 HT scanner and NanoZoomerDigtal Pathology Image System (Hamamatsu Photonics K.K., Hamamatsu, Japan) was used for digital images and snapshots. 21 human UM and 8 UM liver metastasis tissues were included in the study.

Inclusion criteria were as follows: histologically proven UM and availability of formalin-fixed paraffin-embedded tissue samples for IHC. Exclusion criteria were as follows: insufficient specimen for IHC and prior history of brachytherapy or radiotherapy.

### cAMP assay

ACTOne-CXCR4 cells were plated on a 384-well black clear plate at an approximate density of 1.2 × 10^4^ cells/well in 20 µL culture medium. On the third day, the 20 µL of 1 × MP dye solution (Codex BioSolutions Inc, Gaithersburg, MD) was loaded into each well. The cell plate was incubated at room temperature, in the dark, for 2 h. The baseline fluorescent intensity (F0) of each well was recorded on FlexStation.

### Agonist assay

Different concentrations of SDF-1-α (5 × the final concentration) were diluted in 1× DPBS with 0.05% CHAPS, 125 µM Ro20-1724 and 1.5 µM Iso. 10 µL of the solution was added into each well and incubated at room temperature in the dark for a period as indicated. The fluorescent intensity (Ft) of each well was recorded again on FlexStation. The ratios of Ft/F0 were used to plot the dose response curves.

### Antagonist assay

Different concentrations of antagonists (5× the final concentration) were diluted in 1X DPBS. 10 µL of the antagonist solution was added into each well and incubated at room temperature in the dark for 25 min. The baseline fluorescent intensity (F0) of each well was recorded on FlexStation. 10 µl of 6X stimulation solution (60 nM SDF-1-α, 150 µM Ro20-1724 and 1.8 µM Iso in 1× DPBS with 0.05% CHAPS) was then added and incubated at room temperature in the dark for a period as indicated. The fluorescent intensity (Ft) of each well was recorded again on FlexStation. The ratios of Ft/F0 were used to plot the dose response curves.

### Enzyme linked immunosorbent assay (ELISA)

The CXCR4 targeting capability of ProCA32.CXCR4 was measured using an indirect ELISA assay. Briefly, Mel290 uveal melanoma cells were cultured and lysed with RIPA buffer. M20 cell lysate was incubated at 4 °C overnight with NaHCO_3_ solution (pH 9.6) in the 96 well ELISA plates. The ELISA plates were then washed three times with TBST buffer and blocked with 5% BSA solution for 1 h at room temperature. Different concentrations (from 0 nM to 24,000 nM) of ProCA32.CXCR4 solution were prepared in TBST and incubated in different wells of ELISA plate for 1 h at room temperature. After three washes, an in-house ProCA32.CXCR4 antibody was used for incubation at room temperature for 1 h. A stabilized goat-anti-rabbit HRP-conjugated secondary antibody (Catalog number: 34028; ThermoFisher Scientific, Waltham, MA) was then incubated in the wells at room temperature for 45 min. After washing with TBST, 100 µL of 1-StepTM Ultra TMB-ELISA Substrate Solution (Catalog number: G-21234; ThermoFisher Scientific, Waltham, MA) were added into each well for color change visualization. 100 µL of 1 M H2SO4 was added into each well to stop the reaction after observing a gradient color change. The absorbance intensity at 450 nm of each well was measured by FLUOstar OPTIMA plate reader. The binding affinity of ProCA32.CXCR4 to CXCR4 was plotted using KaleidaGraph. Each sample was triplicated, and ELISA was replicated twice.

### Immunofluorescence staining

Immunofluorescence double staining with CXCR4 and gp100 was performed using Opal Automation Multiplex IHC Detection Kits (Akova Bioscience, Inc. Marlborough, MA) in Ventana DISCOVERY ULTRA System (Roche, Tucson, Arizona), according to the manufacturer’s instructions. Briefly, formalin-fixed paraffin-embedded tissue specimens were sliced into 5 µM sections. Tissue sections were deparaffinized and blocked with a blocking buffer, then incubated with the CXCR4 antibody (Catalog number: ab124824; Abcam, Cambridge, MA, 1:400 dilution) for 40 min. Following a thorough wash, the tissue sections were incubated with the secondary antibody and developed with Opal520. Tissue sections were then incubated with anti-melanoma gp100 antibody (Catalog number: ab137078; Abcam, Cambridge, MA, 1:200 dilution) for 40 min. After a thorough wash, the tissue sections were incubated with the secondary antibody and developed with Opal570. DAPI was applied as the nuclear counterstaining. Images were scanned and analyzed with Phenochart TM 1.0 Whole Slide Contextual Viewer for Annotation and Review (Akova Bioscience, Inc. Marlborough, MA)

### MRI scan

OCM1 and OMM2.3 mice were scanned with a 7.0 T Bruker MRI scanner. M20-09-196 mice were scanned with 7.0 T Agilent MRI scanner. MRI scans in OMM2.3 mice were replicated two times. Mice were anesthetized by inhaling isoflurane through an isoflurane vaporizer. Respiration rates of mice were controlled at 50–70 times/min throughout the scanning and recorded every 15 min. T1- and T2-weighted MRI images were acquired with RARE sequence before and after the administration of 100 µL, 5 mM ProCA32.CXCR4. The T1-weighted acquisition parameters were: TR = 500 ms; TE = 10.7 ms; Field of view (FOV), 3.5 cm × 3.5 cm with a matrix of 256 × 256; thickness: 1 mm with no gap. The T2-weighted acquisition parameters were: TR = 2000 ms; TE = 44 ms; FOV, 3.5 cm × 3.5 cm with a matrix of 256 × 256; thickness: 1 mm with no gap.

SKOV3 ovarian cancer mice were scanned with 4.7 T small-bore Varian MRI scanner at Emory University. Mice were anesthetized follow a similar procedure and T2-weighted images were collected with fast spin echo sequence before and 24 h after one bolus injection of 0.025 mmol/kg ProCA32.CXCR4. The fast spin echo sequence parameters were: TR/ESP, 5000 ms/10 ms; FOV, 3.5 cm × 3.5 cm with a matrix of 512 × 512; thickness, 1 mm with no gap. MRI data were processed by Fiji.

### Animal models

All experiments involving the use of animals in this study complied with Association for Research in Vision and Ophthalmology Statement and an approved animal protocol by the Institutional Animal Care and Use Committee (IACUC) at Georgia State and Emory Universities. Five mice were randomly assigned to generate animal models by tumor cells with different levels of CXCR4. Each mouse was scanned by MRI with the control contrast agent ProCA32 as the self-control group before the CXCR4 targeted contrast agent ProCA32.CXCR4.

Metastatic UM mouse models, including M20-09-196, OCM1, and OMM2.3 were developed by intraocular inoculation of corresponding UM cells to 10-wk old female NU/NU mice (Jackson Laboratory, Bar Harbor, ME). More specifically, UM cells were cultured, harvested, and suspended in sterile PBS buffer. Aliquots of approximately 10^6^ UM cells in 2.5 µL of PBS buffer were inoculated into the supra choroid space of the right eye of NU/NU mouse using a transcorneal technique. A mixture of ketamine and xylazine was administered though intraperitoneal injection to anesthetize the mice. A 30 1/2-gauge needle was used to create a tunnel from the limbus within the cornea, sclera and ciliary body to the choroid under a surgical microscope. The tip of a 10 µL glass syringe with a 31-gauge / 45-degree point metal needle (Hamilton, Reno, NV) was used to introduce the cell suspension into the supra choroid space through the needle track. After two weeks of inoculation, the eye was enucleated.

The SKOV3 orthotopic human ovarian cancer xenograft mouse model was developed by Dr. Lily Yang with an established procedure. SKOV3 cells were collected when the confluence reached 80% and suspended in sterile PBS buffer. Female NU/NU mice (Jackson Laboratory, Bar Harbor, ME) were anesthetized by injecting a mixture of 95 mg/kg ketamine hydrochloride and 5 mg/kg xylazine of body weight in sterile saline, intramuscularly. The right ovaries of mice were exposed by abdominal incision. Approximately 5 × 10^4^ cells in 20 μL PBS were injected orthotopically into the ovarian bursa of each mouse. The abdominal incisions were closed after the injection, and mice were monitored until they were completely awake. Primary tumors (size > 6 mm) and metastases were observed to form in the liver, kidney, and spleen after six weeks.

### CXCR4 immune reactive score

All slides were digitally scanned using a NanoZoomer 2.0 HT scanner (Hamamatsu Photonics K.K., Hamamatsu, Japan). CXCR4 positive cells were quantified in digitized images with Aperio ImageScope (Leica, Heinrich-Wild-Strasse, Switzerland). The number of CXCR4 + cells and the number of total cells was counted in six fields with 200× magnification for each specimen. The IRS of CXCR4 was calculated by a semi-quantitative method initially described by Remmele & Stegner for evaluating estrogen receptor in breast cancer tissue and modified by Dobner for evaluating the chemokine receptors on uveal melanoma [23]. The IRS equation is:

$${{{\mathrm{IRS}}}} = {{{\mathrm{staining}}}}\;{{{\mathrm{of}}}}\;{{{\mathrm{tumor}}}}\;{{{\mathrm{cells}}}} \times {{{\mathrm{percentage}}}}\;{{{\mathrm{of}}}}\;{{{\mathrm{stained}}}}\;{{{\mathrm{cells}}}}$$

Staining of tumor cells and percentage of stained cells were scored respectively. The unstained cells score 0, mildly stained cells score 1, moderate stained cells score 2, and strongly stained cells score 3. The scores of percentages of stained cells range from 0 to 4, corresponding to 0%, 0–25%, 25–50%, 50–75% and 75–100% cells.

IRS values were scored by an experimenter blinded to animal model types.

### Statistical analysis

SNR was calculated by the mean value across different metastatic lesions on the MR images of the same mouse. Analyses of differences between the two groups were performed using two-tailed Student’s *t*-test in GraphPad Prism 5 (GraphPad Software). Normality for the dataset was examined by R before conducting *t*-test. The *P*-values are denoted in figure legends, and differences were considered significant if *P* < 0.05. No estimation of sample size and blinding was performed for animal studies. Linear regression plots for the images correlation were conducted with R. Receiver Operating Characteristic (ROC) analyses were performed using R. AUC (area under ROC curve) was reported to measure the performance of the contrast agent. Sample sizes of metastases and SNR were based on estimations by power analysis with a level of 0.05 and a power of 0.9. Data distribution plots including kernel density plots and histograms were made by R.

## Results

### CXCR4 expression level varies in primary tumor, and consistently high in UM metastases

**Fig. 1:**
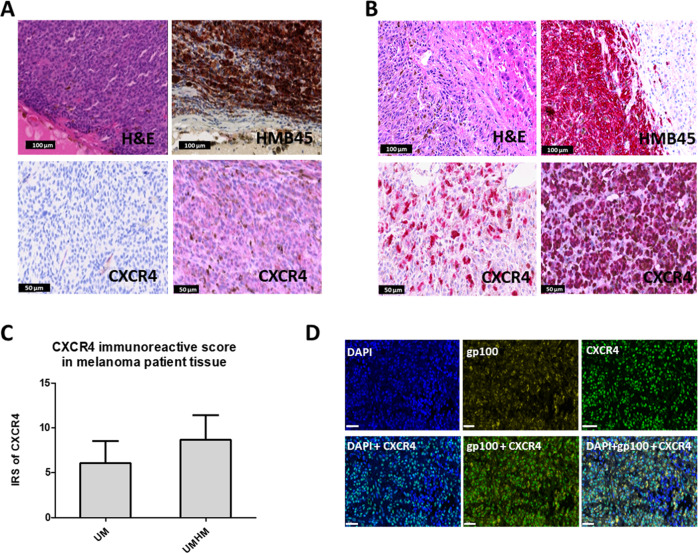
Evaluation of CXCR4 expression levels in primary UM and hepatic metastases of UM (HMUM). **A** Histological analysis of primary UM tissue. H&E staining (top left) shows the morphology of UM, and the diagnosis of UM is confirmed by melanoma marker Human Melanoma Black (HMB45, brown) IHC staining (top right). CXCR4 IHC staining shows low (bottom left) to moderate (bottom right) expression level in UM tissues. **B** Histological analysis of HMUM. H&E (top left) and HMB45 (top right) IHC staining (red) confirmed the lesion as UM metastases in the liver. CXCR4 IHC staining (bottom left and bottom right) shows high expression levels (denoted by red staining) in HMUM. **C** Immunoreactive score (IRS) of CXCR4 in primary UM (UM) and hepatic metastasis of UM (HMUM). HMUM has significantly higher CXCR4 IRS than UM (*P* < 0.01). **D** Immunofluorescence staining of DAPI (blue, top left), gp100 (yellow, top center), and CXCR4 (green, top right) in UM hepatic metastases tissue (scale bars, 60 µm). The spatial overlapping of gp100 and CXCR4 (bottom center) demonstrated CXCR4 expressed on tumor cells (scale bars, 60 µm). Bottom left: the merged image of CXCR4 and DAPI; bottom right: the merged image of CXCR4, gp100 and DAPI.

The CXCR4 expression in primary and metastatic UM patient tissues was analyzed using immunohistochemistry (IHC) staining. CXCR4 expression was categorized into low (below 30%), intermediate (30–60%), and high level (above 60%) based on the percentage of positively stained cells and evaluated by digital processing. Study results for 21 UM specimens found 8 (38.1%) with high level, 9 (42.9%) with intermediate level, and 4 (19.1%) with low level of CXCR4 expression. In hepatic metastases from UM, 8/8 (100%) show a high level of CXCR4. The CXCR4 immunoreactivity of UM tissues was also scored using an immunoreactive score (IRS) determined by multiplying the percentage of positively stained cells by the intensity of staining. An IRS score less than 4 is defined as low level expression, a score between 4–8 was intermediate level expression, and greater than 8 was high expression. The CXCR4 expression level varies in 21 primary UM tissues: 7 (33.3%) with high level, 8 (38.3%) with intermediate levels, and the remaining 6 (28.6%) with low level of CXCR4 expression (Figs. 1A and S1). However, all 8 cases of UM liver metastases were found to express high levels of CXCR4 (Fig. 1B and C and Fig. S1). Statistical analysis showed that the IRS scores of CXCR4 expression in liver metastases from UM were significantly higher than those of primary UM tumors (*P* < 0.01, Fig. 1C). We further performed immunofluorescence staining on hepatic UM metastases to analyze the spatial relationship of CXCR4 expression and tumor cells (Fig. 1D). Melanoma cells were stained using glycoprotein 100 (gp100), denoted by yellow fluorescence. The CXCR4, indicated by green fluorescence, was distributed mostly on the melanoma cells.

**Fig. 2:**
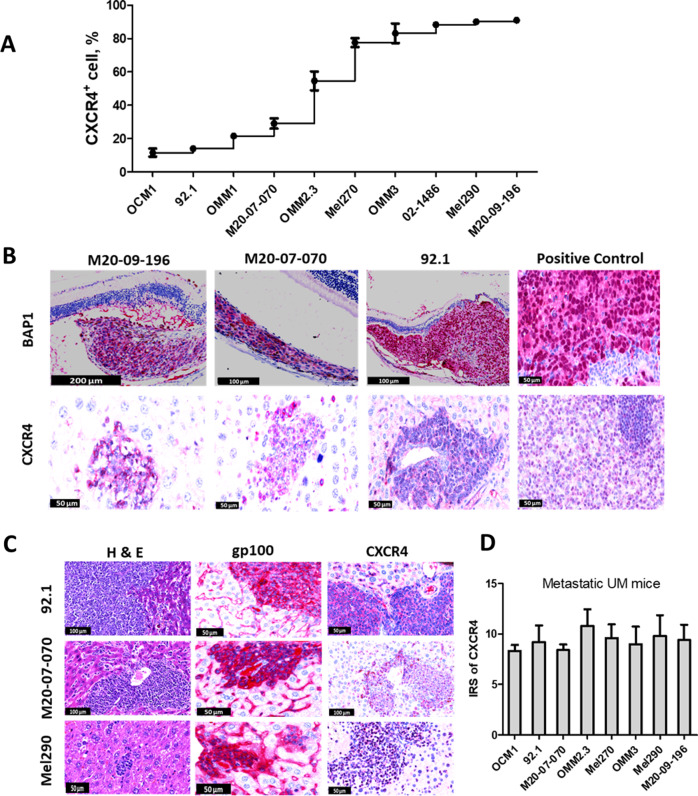
CXCR4 expression level in vitro and in liver metastases (murine model). **A** Flow cytometry analysis of CXCR4 expression in UM cell lines. The CXCR4 expression of ten different UM cell lines were evaluated and the expression varied from 11.5% (OCM1) to 91% (M20-09-196). **B** BAP1 expression (red, top row) in UM: no nuclear BAP1 in M20-09-196 and M20-07-070 or nuclear BAP1 expression in 92.1 and positive control (human UM), CXCR4 expression presented in their hepatic metastases of UM mouse models and tonsil as a positive control (bottom). **C** CXCR4 expression was found in cytoplasm of liver metastases (right column) in UM mouse models generated by 92.1 (top), M20-07-070 (middle) and Mel290 (bottom), which expressed low, intermediate, and high levels of CXCR4 in vitro, respectively. Histology (left column) and gp100 (middle column) identified the formation of hepatic metastases from ocular melanoma. **D** Quantitative analysis showed high levels of CXCR4 expression in hepatic metastases in UM mouse models generated by eight types of human UM cell lines.

Next, we examined the levels of CXCR4 expression in 10 different UM cell lines using flow cytometry. The results showed that CXCR4 expression varied from low to high across different cell lines (Fig. 2A). CXCR4 expression was low in OCM1 (11.6 ± 2.5%), 92.1 (14.0 ± 0.6%), and OMM1 (21.5 ± 0.7%), intermediate in M20-07-070 (29.1 ± 3.1%) and OMM2.3 (54.6 ± 5.7%), and high in Mel270 (77.6 ± 2.7 %), OMM3 (83.2 ± 5.8%), 02-1486 (88.4 ± 0.5%), Mel290 (90.2 ± 0.4%), and M20-09-196 (91.0 ± 1.3%). It was clear that CXCR4 expression levels differed across different UM cell lines consistent with histological studies indicating high heterogeneity in primary UM.

### High level of CXCR4 persistently expresses in liver metastases of UM murine models

**Fig. 3:**
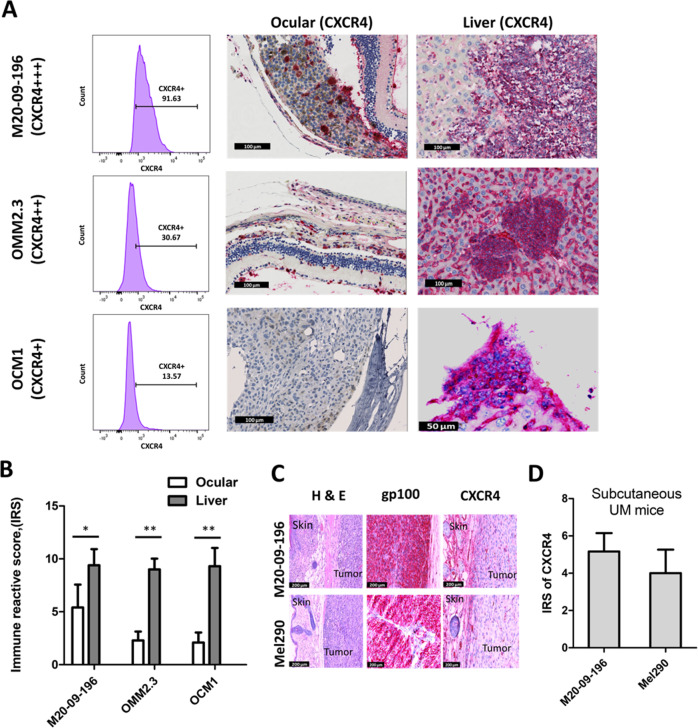
Comparison of CXCR4 expression levels between liver metastatic murine models and subcutaneous mouse models. **A** In vitro UM cell lines with high (+++, 91.6% positive, top left), intermediate (++, 30.7% positive, middle left), and low (+,13.6% positive, bottom left) expression levels of CXCR4 by flow cytometry were used for generation of metastatic UM mice models. The CXCR4 expression levels obtained by IHC in primary eye UM was intermediate in M20-09-196 mice (top middle), and low in OMM2.3 (middle) and OCM1 mice (bottom middle). The CXCR4 expression levels by IHC in hepatic metastases was high in all three mice models (top right, middle right and bottom right) regardless of the CXCR4 expression levels in vitro and in primary ocular UM. **B** Comparison of CXCR4 IRS between ocular UM and UM hepatic metastases from M20-09-196, OMM2.3, and OCM1 mice models. The CXCR4 expression (represented by IRS) was significantly elevated in hepatic UM in comparison with the primary ocular UM in M20-09-196 (*p* < 0.05), OMM2.3 (*p* < 0.01), and OCM1 mice models (*p* < 0.01). **C** H&E staining (left), gp100 IHC staining(middle), and CXCR4 staining (right) of subcutaneous UM tumors generated from M20-09-196 and Mel290 cells. **D** Quantitative comparison of CXCR4 IRS between subcutaneous UM tumor from M20-09-196 and Mel290.

To understand the relationship between prognostic biomarkers BAP1 in primary tumors and CXCR4 in liver metastases, both the expression of BAP1 [10, 18, 19] and CXCR4 were evaluated in murine models. A suprachoroidal inoculation of different UM cell lines were performed in the nude mice, and metastases gradually formed in the liver of the mice over time. The cell lines used for construction of metastatic UM murine models including M20-09-196, M20-07-070, 92.1 and Mel290. Each of the cell lines carries a different BAP1 genotype. M20-09-196 cells show low level of BAP1, loss of BAP1 expression in nuclei due to a large in-frame deletion in the enzymatic domain of BAP1; M20-07-070 cells exhibit an intermediate level of BAP1, a nonsense mutation in the NLS domain of BAP1 (Q342X) caused the loss of expression; 92.1 with a high level of BAP1 exhibit BAP1 expression in nuclei; and Mel290 cells exhibit nuclear BAP1 expression in intraocular tumors due to the wild-type BAP1 gene [24]. Despite their differences in BAP1 expression, all four cell lines generated liver metastases with significantly higher CXCR4 expression than their in vitro level (Fig. 2B and C). Additionally, chromosomal aberrations like monosomy 3 are associated with a poor prognosis in UM patients [10]. M20-07-070 cells with monosomy 3 as well as 92.1 and Mel290 cells with disomy 3 [24, 25] expressed high levels of CXCR4 in their liver metastases (Fig. 2C, D). Guanine nucleotide-binding protein G(q) subunit alpha (GNAQ) mutations indicate initial events in tumorigenesis and specific drug responses [10]. However, cells with GNAQ mutations (M20-09-196, M20-07-070, 92.1) and cells with the wild-type gene (Mel290, OCM1) [24–26] both expressed high levels of CXCR4 in their liver metastases (Fig. 2B and C; Fig. 3A, B). Therefore, regardless of variables such as BAP1 expression, monosomy 3 and GNAQ mutations, CXCR4 functioned as an independent biomarker of hepatic metastasis.

Metastatic models generated by UM cells 92.1, M20-07-070, and Mel290, that expressed low, medium, and high levels of CXCR4 in vitro, all displayed high CXCR4 expression in hepatic metastases (Fig. 2C and D). CXCR4 expression was further detected in all possible UM hepatic metastases using additional metastatic UM murine models constructed from 3 additional UM cell lines: OMM3, OMM2.3 and Mel270 (Fig. S2). Subsequent hepatic metastases from all 8 UM murine models demonstrated high levels of CXCR4 expression, indicated by a mean value of the IRS over 8 (Fig. 2D). Fig. 3 shows that the CXCR4 expression of UM cells changes with environment. Cultured UM cell lines M20-09-196, OMM2.3, and OCM1 expressed high, medium, and low levels of CXCR4 in vitro, demonstrated by the FACS (Fig. 3A, left column). When inoculated to the eyes, the intraocular tumors generated from OCM1 and OMM2.3 displayed low levels of CXCR4 expression, whereas the M20-09-196 intraocular tumors displayed medium levels of CXCR4 expression (Fig. 3A). However, when metastasized to the liver, the metastases developed from all three UM cell lines are all showing high level of CXCR4 expression (Fig.  3A, right column). The IRS scores of intraocular M20-09-196, OMM2.3, and OCM1 tumors were 5.4 ± 2.7, 2.3 ± 0.8, and 2.1 ± 0.9 (Fig. 3B). In contrast, the IRS values for hepatic metastases of these three models were 9.4 ± 1.5, 9.0 ± 1.0, and 9.3 ± 1.7, which were significantly higher than the intraocular IRS value (*p* < 0.05, Fig. 3B). Regardless of CXCR4 expression level in cultured UM cells and intraocular tumors using these cell lines in our mouse models, the hepatic metastases always exhibited higher levels of CXCR4 than the intraocular tumors. Our results indicated that CXCR4 expression in UM is upregulated in hepatic metastases.

### Subcutaneous UM murine models exhibit decreased CXCR4 expression

To further clarify whether upregulation of CXCR4 resulted from different tumor microenvironments, two additional subcutaneous UM murine models were generated using UM cell lines M20-09-196 and Mel290. These two UM cell lines both expressed high levels of CXCR4 in vitro (Fig. 2A). However, when inoculated subcutaneously, both UM cell lines developed tumors that exhibited medium levels of CXCR4 expression (Fig. 3C) with the IRS values for M20-09-196 and Mel290 being 5.2 ± 1.0 and 4.0 ± 1.3, respectively. The primary tumors established by subcutaneous inoculation exhibited significantly decreased expression of CXCR4 compared with their parental cells. Additionally, a decrease of CXCR4 expression in UM tumors was observed in the subcutaneous environment. This was in sharp contrast to results observed with liver metastases and was likely due to lack of high gradient of ligand CXCL12. Thus, our studies demonstrated a consistent and significant increase of CXCR4 expression in liver metastasis, confirming CXCR4 can serve as a biomarker for detection of hepatic metastasis.

### Protein based MRI contrast agent with high relaxivity and CXCR4 binding affinity

**Fig. 4:**
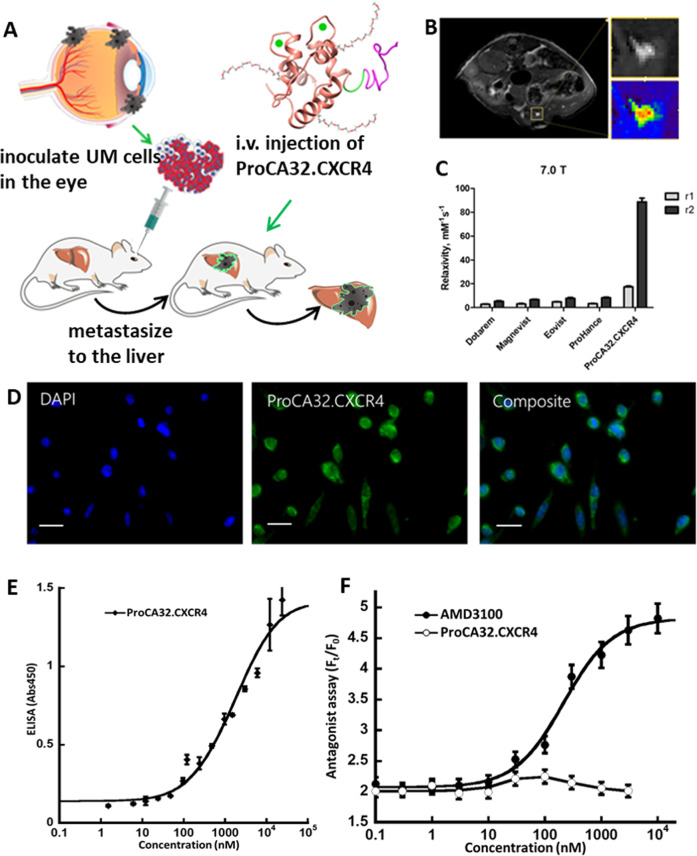
Development of CXCR4 targeted contrast agent ProCA32.CXCR4 for CXCR4 molecular MR imaging. **A** The working scheme of ProCA32.CXCR4 imaging UM liver metastases in the mouse models. UM cell lines were derived from UM patient tissue. After inoculation of the UM cells in the eye of the mice, metastatic lesions will form in the liver over time. ProCA32.CXCR4 administered through I.V. injection. **B** Intensity enhancement of UM liver metastatic lesions by MRI molecular imaging. **C** Relaxivity measurement of ProCA32.CXCR4 and clinical counterparts including Dotarem, Magnevist, Eovist, and ProHance. At 7.0 T magnetic field, ProCA32.CXCR4 exhibited notably higher r1 and r2 values compared to the other gadolinium-based clinical contrast agents. **D** CXCR4 targeting study of ProCA32.CXCR4 through immunofluorescence staining. Blue fluorescence is the DAPI staining of nucleus. Green fluorescence indicates the fluorescein labeled ProCA32.CXCR4 on Mel290 cells, scale bar, 10 µm. **E** Quantitative measurement of CXCR4 targeting capability of ProCA32.CXCR4 by ELISA. The K_d_ value of ProCA32.CXCR4 binding to CXCR4 was 1.19 ± 0.28 µM. **F** Determination of CXCR4/CXCL12 mediated downstream activity using cAMP assay. Binding of ProCA32.CXCR4 to CXCR4 does not produce any significant cAMP activity, which is very different from antagonist AMD3100.

We have developed a platform technique to achieve molecular imaging of different biomarkers such as collagen 1 [27, 28], prostate-specific membrane antigen (PSMA) [29] etc. To achieve CXCR4 targeted molecular MRI imaging, we incorporated a CXCR4 targeting moiety into a protein scaffold ProCA32 [30], and ProCA32.CXCR4 [31] (Fig. 4A). ProCA32.CXCR4 exhibits high r1 and r2 values at both low and high magnetic field, the values are 6 to 20 times higher than other Gd^3+^ based clinical contrast agents. The CXCR4 targeting capability and improved relaxivity of ProCA32.CXCR4 enables the molecular MRI in detecting liver metastases (Fig. 4B). At 7.0 T, the r1 and r2 value were of 17.4 and 88.7 mM^−1^s^−1^ (Fig. 4C). ProCA32.CXCR4 has been shown to bind to overexpressed CXCR4 on cancer cell lines such as M20-09-196 and Mel290, using immunofluorescence staining (Fig. 4D). It exhibits strong binding affinity to overexpressed CXCR4 in cancer cells with a Kd of 1.19 µM, determined using Elisa (Fig. 4E). On the other hand, binding of ProCA32.CXCR4 to CXCR4 does not produce any significant cAMP activity, which is very different from results of the approved drug AMD3100 (Fig. 4F). Thus, ProCA32.CXCR4 binds to CXCR4 without the complication of triggering downstream signaling, a property that makes it highly desirable as an imaging agent for diagnosis and following treatment effects.

### Early detection of liver metastases by molecular MRI

**Fig. 5:**
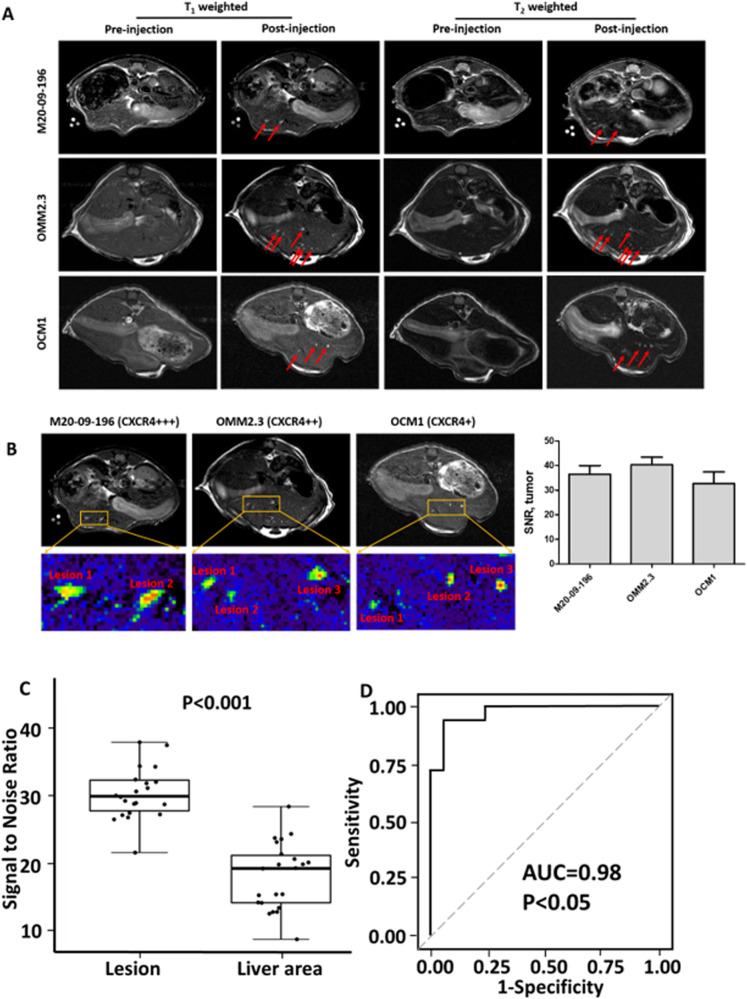
MR images of metastatic mice models with ProCA32.CXCR4 administration. **A** Comparison of MRI images of metastatic mice models including M20-09-196, OMM2.3, and OCM1, before and after administration of ProCA32.CXCR4. Both T1-weighted (left two columns) and T2-weighted (right two columns) MR images showing metastatic lesions, illuminated following the administration of ProCA32.CXCR4. Red arrows point to the UM metastases in the liver. **B** Zoom-in view of the metastases from M20-09-196, OMM2.3, and OCM1 mouse models; MRI signal-noise-ratio (SNR) of metastases following ProCA32.CXCR4 administration. **C** The box-and-whisker plot of tumor lesion SNR and liver SNR. The p-value of less than 0.001 generated from student’s *T*-test indicates a significant difference between tumor SNR and liver SNR. **D** ROC plot with statistical analysis that suggests ProCA32.CXCR4 provides diagnostic validation for UM metastases in the liver (lesions *n* = 22, mice *n* = 4). *P* < 0.05 indicates the significance.

**Fig. 6:**
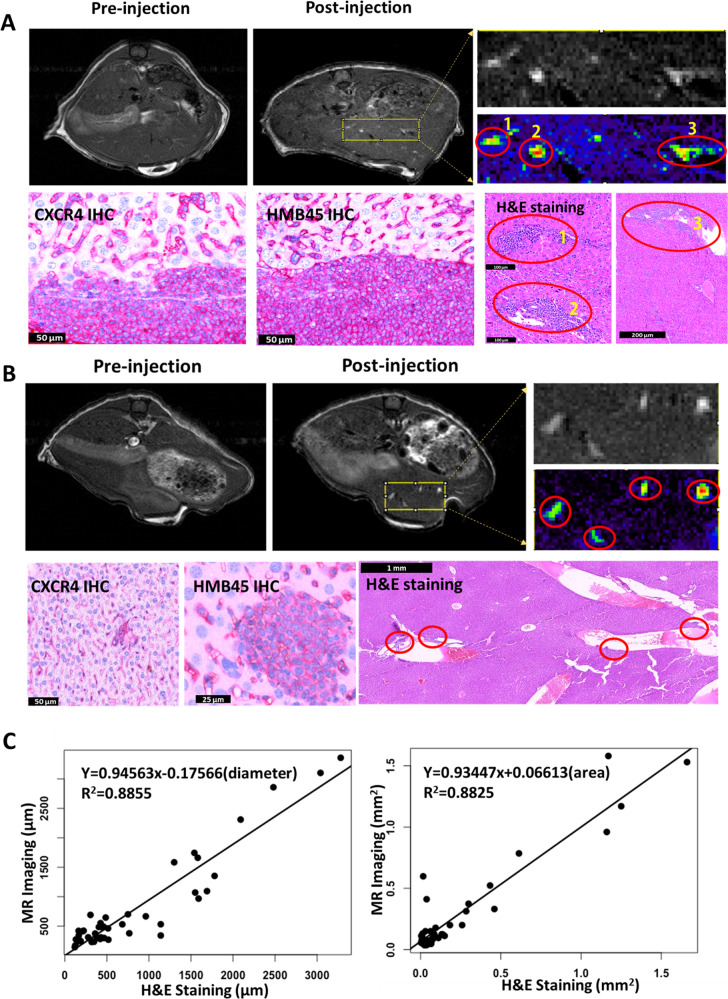
MR images and histological correlation of hepatic metastases in mice models OMM2.3 and OCM1 with ProCA32.CXCR4 administration. **A** MR image of OMM2.3 mouse following the administration of ProCA32.CXCR4 (post-injection) shows the metastatic lesions in the liver. The metastasis in the MR image was identified in the H & E staining slide of the liver tissue from the same mouse. Immunofluorescent staining of the tumor tissue verified the expression of CXCR4 (green) and gp100 (yellow), CXCR4 and gp100 expressions are overlapped (merge). **B** Post-injection MR image of the OCM1 mouse exhibits the liver metastases that are invisible in the pre-injection MR image. Metastases in the H&E staining are well-correlated with the ones recognized in the MR image and show positive during CXCR4 and HMB45 immunohistochemistry staining. **C** The measurement of metastases (*n* = 45) in MR images correlates with the corresponding results in H&E staining, both in diameters (left, *Y* = 0.945 *x*−0.176, *R*^2^ = 0.885) and areas (right, *Y* = 0.934 *x* + 0.066, *R*^2^ = 0.882).

We examined if high r1 and r2 relaxivity values and strong CXCR4 targeting capability enable detection of early stage of various types of liver metastasis in vivo using our established animal models (Fig. 5A). We performed MRI imaging of liver metastases using three UM murine models with administration of ProCA32.CXCR4. These murine models generated by OCM1, OMM2.3, and M20-09-196 UM cell lines, with low, medium, and high CXCR4 expression levels in vitro. Results demonstrated that tail-vein injection of 0.025 mmol/kg of ProCA32.CXCR4 enabled detection of these metastases in all three models using both T1 and T2 weighted MR imaging (Fig. 5A and B and Fig. S3). In contrast, no hepatic metastases were visible in the pre-injection MR images and could not be detected using clinical contrast agent Eovist or the non-targeted MRI contrast agent ProCA32 (Fig. S4). Detected liver metastases were then verified by histological analysis in the OMM2.3 mouse model. H&E staining verified the metastases with corresponding space-arrangement to the MR images (Fig. 6A). Four metastatic lesions in OCM1 liver H&E staining were found to correlate spatially with the metastases enhanced in the MR images (Fig. 6B). Results of MRI were further confirmed to be melanoma metastases by HMB45 IHC staining. IHC staining for CXCR4 expression was positive. The diameters and areas of lesions in MR images correlated very well with the corresponding measurements in H&E staining of tissue sections (Fig. 6C).

The size distribution of all detected and verified lesions by MRI remains similar to that by histological analysis despite difference in resolution (Fig. S5). Among the lesions validated by histological analysis in all three models, the majority of tumor diameter were between 0.2 mm to 0.5 mm and with most of the 45 tumor diameters falling below 1.0 mm as seen in its statistical distribution (Fig. S5). The liver metastases’ diameter in the 0.2–0.5 mm range is defined as Stage 2 of liver metastasis (36), which indicates the transition phase from a dormant status to an activated status. The metastases from three mice models displayed similar enhancement with no significant differences observed for the signal-noise-ratio (SNR) of the metastases from OCM1, OMM2.3, and M20-09-196 mice (*p* > 0.05). However, a significant difference in SNR was detected between tumor lesions and liver tissue (*p* < 0.001) (Fig. 5C) with an area under curve (AUC) of 0.98 (*p* < 0.05) (Fig. 5D), suggesting that MRI results can readily differentiate these early-stage tumors from healthy liver tissue. These in vivo studies validate CXCR4 as an imaging biomarker for liver metastasis. The early detection of uveal melanoma hepatic metastases is achieved by CXCR4 molecular MRI imaging.

### Evaluate CXCR4 expression of hepatic metastases in an ovarian cancer murine model

**Fig. 7:**
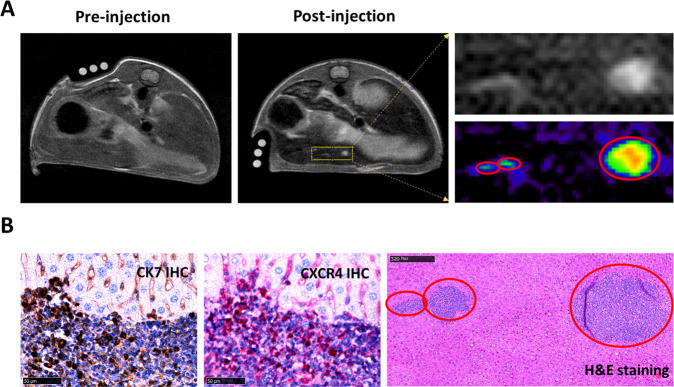
Detection of liver metastases from ovarian cancer through CXCR4 targeted MR imaging. **A** MR image of ovarian cancer mouse model (SK-OV-3) shows the enhancement of liver metastatic lesions with ProCA32.CXCR4 administration. **B** Metastases in the MR image were identified in the H&E staining (red-circle regions) and proved to be positive in CK7 (brown) and CXCR4 (red) IHC staining.

In order to evaluate CXCR4 expression in an additional hepatic metastatic mouse model, we used human ovarian cancer, distinct from UM, and verified this with MRI using ProCA32.CXCR4. Since CXCR4 expression has been identified as an independent prognostic factor for ovarian cancer patients [32], and inhibitors of CXCR4 improved overall survival of mice with metastatic ovarian cancer [33]. An ovarian cancer xenograft mouse model that was generated by the human ovarian adenocarcinoma SK-OV-3 cell line and subsequently appeared as liver metastases, was used to evaluate the putative upregulation of CXCR4 expressed in this additional murine model of another type of cancer. MRI with ProCA32.CXCR4 confirmed CXCR4 expression in liver metastases, and ProCA32.CXCR4 enabled the detection of liver metastasis lesions ranging from 0.02 mm^3^ to 0.54 mm^3^ in SK-OV-3 mice. The adenocarcinoma marker cytokeratin-7 (CK7) and CXCR4 IHC staining further verified the liver lesions as adenocarcinoma that exhibited CXCR4 expression. The spatial arrangement of MRI-detected metastases matched with that of H&E staining of the liver tissue (Fig. 7). Taken together, these studies further validate CXCR4 as an imaging biomarker of hepatic metastasis. The detection of liver micrometastases in an ovarian adenocarcinoma murine model was demonstrated by the CXCR4 targeted MRI imaging.

## Discussion

To achieve early detection of liver metastases using molecular imaging, it is essential to identify a molecular biomarker with consistently high expression in the liver metastases. Previous studies reported that mutations in the tumor suppressor BAP1 are associated with liver metastases in UM patients [19], and lack of UM nuclear BAP1 immunoreactivity has been proposed as an indicator of metastatic potential. However, some UM with wild type BAP1 or nuclear BAP1 still develop metastasis [15, 18]. Contrastingly, our studies here revealed that CXCR4 always expresses in liver metastases from UM patients and various UM mouse models, no matter the BAP1 or other prognostic factors like chromosomal aberrations in primary UM. Because of this, the CXCR4, not BAP1, is suitable as an imaging biomarker.

CXCR4 has been shown to play a crucial role in liver specific metastases formation due to the high expression of its natural ligand CXCL12 (SDF-1) in the liver. The dual blood supply in the liver, the anatomy of sinusoidal spaces, and the high CXCL12 expressing sinusoidal endothelial cells together facilitates circling tumor cells with CXCR4 expression adhere and extravasate into the liver parenchyma and establish distant organ metastases [34]. CXCR4 overexpression in primary cancers is associated with poor prognosis [35, 36]. Figueras et al. reported the higher CXCR4 levels in high-grade serous epithelial ovarian carcinomas (the most metastatic tumors), in comparison with those in endometrioid carcinomas [20]. In an immunohistological analysis of 103 patients with pancreatic cancer, strong CXCR4 expression was significantly correlated with advanced pancreatic cancer and reveals a trend for hematogenous metastasis [37]. Treatment by an agent which inhibits CXCR4 or a CXCR4 antagonist can reduce liver metastases [22, 38]. These imply that CXCR4 likely is a biomarker of liver metastasis.

In this study, we demonstrated that high expression of CXCR4 is a molecular biomarker for liver metastases in both patient tissues and animal studies, despite its variable expression in primary tumors. We analyzed the CXCR4 expression of UM cell lines in vitro using flow cytometry and evaluated the expression in UM tumors with IHC staining. Our results showed the CXCR4 protein expression for the same cell line varies in vitro and in vivo, and the in vitro level of CXCR4 protein in cell lines has no correlation with the expression level in hepatic metastases. Therefore, we did not further explore the level of CXCR4 mRNA in cell lines. Further quantification of CXCR4 expression in liver metastases from UM murine models constructed by intraocular inoculation of eight UM cell lines with differential expression of CXCR4 demonstrated that the hepatic metastases always exhibited higher levels of CXCR4 than their parent cells and intraocular tumors. The consistently high CXCR4 expression in the liver metastases is independent of multiple prognostic factors, such as BAP1 mutations and monosomy 3. We have observed high CXCR4 expression in liver metastases from murine models generated by cell lines M20-09-196 and M20-07-070, these two cell lines carries different BAP1 mutations [24, 25]. We further demonstrated no increase of CXCR4 expression in the subcutaneous environment using subcutaneous UM murine models (Fig. 3C), which is likely due to the low expression of CXCL12. Our findings suggest the importance of the liver environment in which provided with high expression of the CXCR4 ligand by hepatic sinusoidal endothelial cells and hepatic stellate cells. Consistent with our findings, Li et al reported that the addition of CXCL12 and liver mimic solution increased CXCR4 expression of UM cell lines. A greater liver metastatic number/burden in mice was also observed using CXCR4-positive uveal melanoma cells compared with using CXCR4-negative uveal melanoma cells injected into mice [39]. Unlike the liver, with a high SDF1 concentration, ocular microenvironmental factors induce methylation of unmethylated CpG regions in the CXCR4 promoter and this may contribute to the low expression of CXCR4 [39]. Our results are leading to the conclusion that CXCR4 is an imaging biomarker for detection of liver metastases in vivo.

Previous studies have reported that several cancers including UM, breast cancer, and CRC cancers, share similar liver metastatic growth patterns, despite originating from different primary sites, and metastasizing to the liver via different routes [40–44]. Liver metastases with different pathological growth patterns were speculated to have very different responses to systemic therapy due to their different origins and their differences in collagen expression and angiogenesis [40]. However, growth patterns of radiologically identified liver metastases as determined by currently approved liver MRI contrast agents Eovist® or Multihance® are largely inconsistent with pathologically defined patterns [42–44] due to their inability to detect early stage tumors (<1 cm) with required sensitivity and specificity. All clinical Gd3+ MRI contrast agents have relaxivity (r1) values of approximately 5 mM^−1^ s^−1^, which is significantly lower than the theoretically achievable value [45]. The low relaxivities of commercially available MRI contrast agents largely limit their applications in molecular imaging of biomarkers, especially for receptors whose low expression level are usually around sub µM or nM in vivo. To date, there is no non-invasive methodology for characterizing the role of key molecular regulators during the metastatic progression in the liver.

In this study, we report our development of a protein MRI contrast agent (ProCA32.CXCR4) which can bind to CXCR4 expressed in cancer cells and liver metastases. ProCA32.CXCR4 also exhibits significantly increased r1 and r2 relaxivity at both low and high magnetic fields compared to other clinically approved contrast agents. We report that application of ProCA32. CXCR4 notably improves the detection limit of current imaging methodologies of late-stage metastases (4 out 4, tumor diameter 1–2 cm) to early-stage metastases (stage 2, tumor diameter 0.1 mm) (Figs. 6 and 7 and Fig. S5). Liver metastasis can be designated into different stages based on tumor size: stage 1 metastases, defined as tumor clusters ≤50 μm in diameter; stage 2 metastases, defined as tumors measuring 51–500 μm in diameter; and stage 3 metastases, defined as tumors measuring >500 μm in diameter. Stage 1 metastases are avascular and lacked mitotic activity in a dormant status. The mean vascular density and mitotic index increased from stage 2 to stage 3 metastases. The architecture of stage 2 metastases mimicked the surrounding hepatic parenchyma, whereas stage 3 metastases exhibited either lobular or portal growth patterns. This indicates that stage 3 metastases are activated, and stage 2 metastases are transitioned from stage 1 to stage 3. During this progression, tumors become vascularized and mitotically active [40]. We have demonstrated the early detection of stage 2 metastases using three different metastatic UM models and an ovarian cancer model with administration of ProCA32.CXCR4 (Figs. 5, 6 and 7). In contrast, these small liver metastases cannot be detected by the clinically approved contrast agent Eovist (fig. S3). The specific detection of small liver metastasis by ProCA32. CXCR4 was further verified using histological analysis (Figs. 5 and 6). CXCR4 targeting capability of ProCA32.CXCR4 significantly improved detection sensitivity since ProCA32 with the targeting moiety was not able to detect the small liver metastasis (Fig. S3). Specific binding to the CXCR4 receptor expressed in the tumor was validated as pre-injection of the CXCR4 binding moiety was able to block the corresponding MRI enhancement at the tumor using ProCA32.CXCR4 [31]. We have demonstrated the general applicability of our developed ProCA32.CXCR4 in non-invasive early detection of liver metastasis in two types of cancer. Although the resolution of MRI is lower than microscopic histological analysis, detailed correlation analysis revealed that our non-invasive MRI enabled by proCA32.CXCR4 is able to capture liver metastasis burden with the size distribution very similar to that by histological analysis (Fig. S5). Our work reports the first achievement using non-invasive MRI to capture stage 2 of liver metastasis (diameter 0.1–0.5 mm range) transiting from a dormant status to an activated status [40] with strong sensitivity and specificity in three animal models.

Plerixafor (AMD3100) is an FDA approved antagonist of CXCR4, binding to CXCR4 and inhibiting CXCR4/SDF1 interactions without cross-reactivity with other chemokine receptors [46–48]. AMD3100 has been showed to delay CXCR4-mediated metastasis and invasion of ovarian cancer and reduce self-renewal and survival in human glioblastoma stem-like cells [49]. Blockade of CXCR4 by AMD3100 coupled with the cytotoxic drug dacarbazine significantly inhibited tumor growth and metastasis of melanoma compared to dacarbazine alone [50]. CXCR4 antagonists also suppressed metastatic progression and decreased the number of hepatic micrometastases in an orthotopic mouse model of UM [38, 51]. In a recent phase II trial, a CXCR4 antagonist demonstrated early promise as an agent that enhanced the benefit of chemotherapy for metastatic PDAC when used in combination with a PD-1 inhibitor. For cancer patients exhibiting high CXCR4 expression, the ability to target CXCR4 may be a promising approach to new therapies. Therefore, non-invasive CXCR4-specific monitoring in vivo CXCR4 expression of metastases or as a complementary diagnostic test is also critical for treatment stratification.

Our developed ProCA32.CXCR4 does not alter the down-stream CXCL12/CXCR4 axis signaling with cAMP production, possibly due to a different binding mode [52]. Results of this study further demonstrated that in vivo application of ProCA32.CXCR4 does not result in tissue or organ toxicity [31]. ProCA32.CXCR4 also exhibited high metal selectivity for Gd over physiological metal ions and strong serum stability. These results suggest that ProCA32.CXCR4 has strong translational potential, and we are currently working toward reducing in vivo retention time for improved diagnostic applications.

In summary, we have identified CXCR4 as a molecular biomarker of hepatic metastases using both patient tissues and murine models. We have developed a CXCR4-targeted MRI imaging contrast agent that enables the early detection of small stage 2 liver metastases transiting from a dormant status to an activated status in multiple metastatic murine models using MRI. Further development of our novel CXCR4 MRI contrast agent ProCA32.CXCR4 is expected to have additional applications in following high-risk patients, stratifying personalized treatment, and monitoring treatment efficacy.

## Supplementary information

SUPPLEMENTARY MATERIALS

## Data Availability

All data are available in the main text or the supplementary materials.
