# Supplementary material for: Non-invasive detection and complementary diagnosis of liver metastases via chemokine receptor 4 imaging
Source: Cancer Gene Ther. 2022 Feb 10;29(12):1827–39. doi: 10.1038/s41417-022-00433-w (PMC9363530; doi:10.1038/s41417-022-00433-w)
Supplement: Supplementary file 1 — SUPPLEMENTARY MATERIALS [file 41417_2022_433_MOESM1_ESM.docx]

**SUPPLEMENTARY MATERIALS**

Fig. S1. H&E, HMB45, and CXCR4 staining of UM patient tissues.

Fig. S2. CXCR4 and H&E of hepatic metastases

Fig. S3. MR images of mice with ProCA32 and Eovist injection.

Fig. S4. Post-injection MR image of the OCM1 mouse exhibits the liver metastases which are invisible in the pre-injection MR image.

Fig. S5. UM liver metastases size (tumor diameter) distribution.


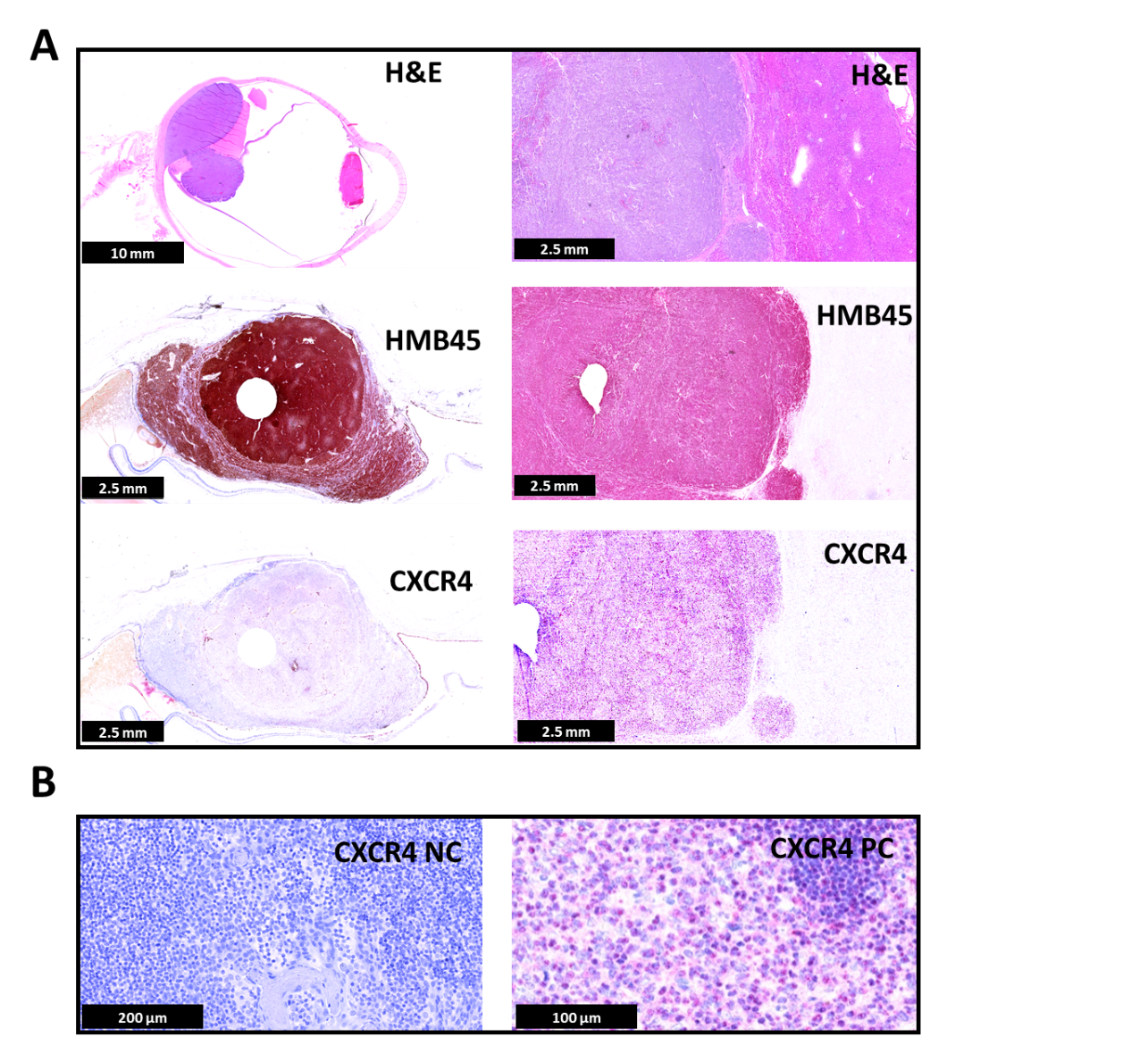


**Figure. S1. H&E, HMB45, and CXCR4 staining of UM patient tissues.** (**A**) H&E, HMB45, and CXCR4 staining of primary UM (left column) and UM liver metastases (right column). (**B**) CXCR4 negative control (NC, left) and positive control (PC, right).


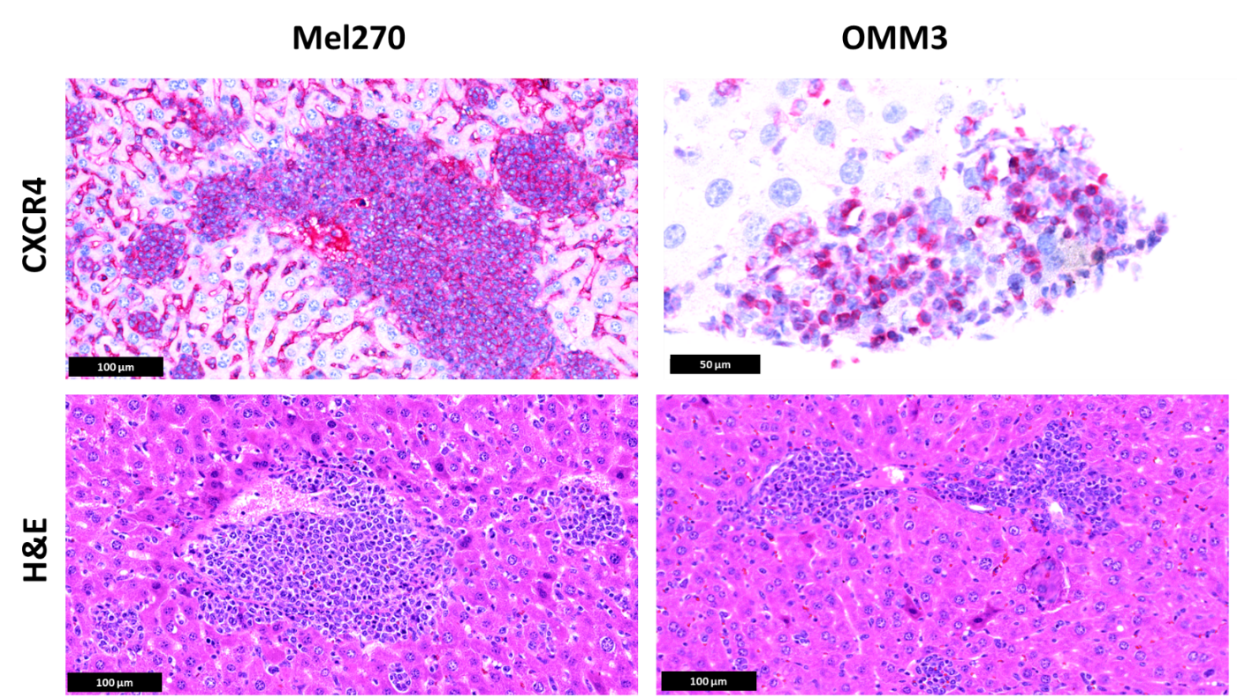


**Figure. S2. CXCR4 and H&E of hepatic metastases from Mel270 (left) and OMM3 (right) UM murine models.**


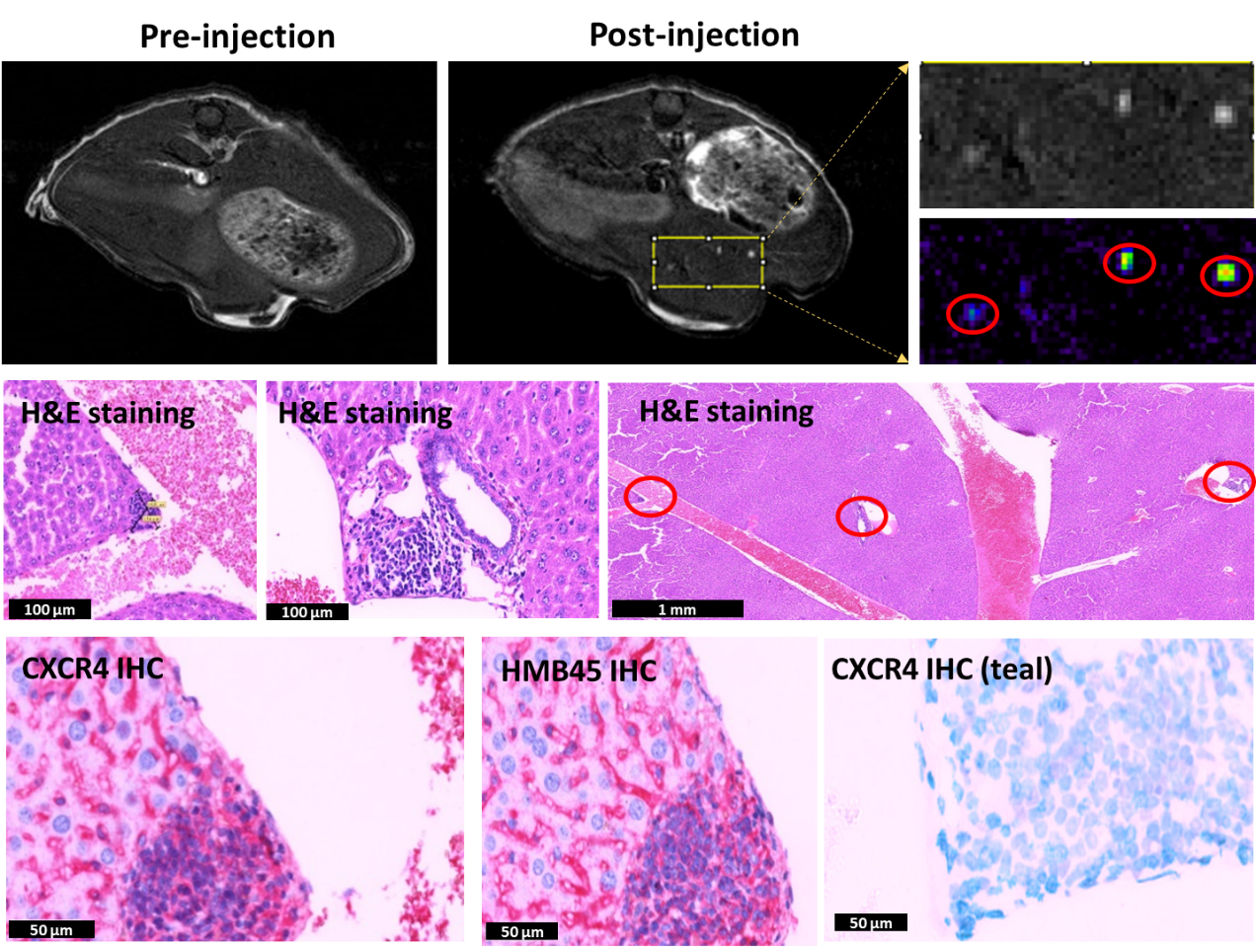


**Figure. S3. Post-injection MR image of the OCM1 mouse exhibits the liver metastases which are invisible in the pre-injection MR image.** Metastases in the H&E staining are well-correlated with the ones recognized in the MR image and proved to be positive for CXCR4 and HMB45 immunohistochemistry staining.


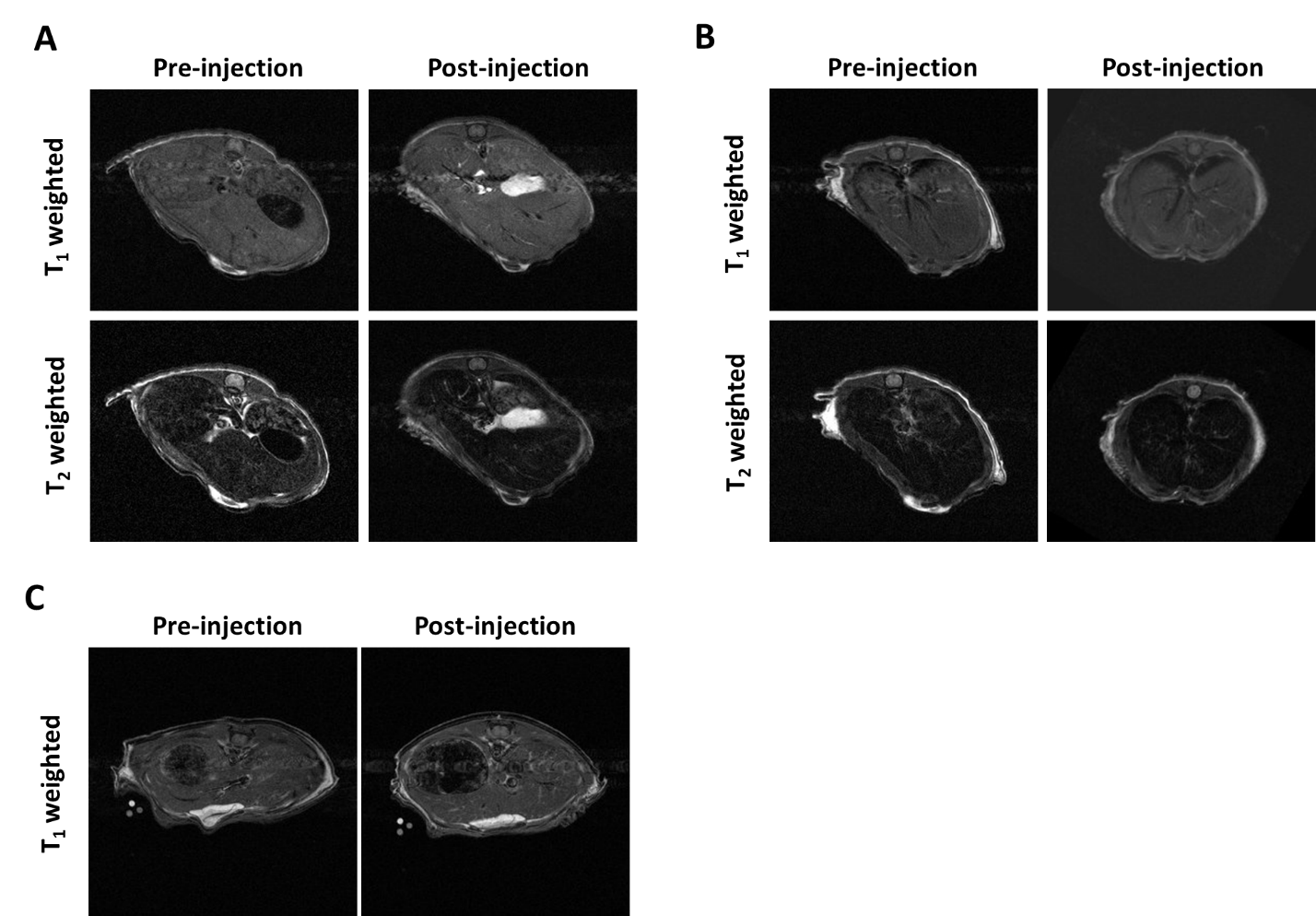


**Figure. S4. MR images of mice with ProCA32 and Eovist injection.** (**A**) T1 weighted and T2 weighted MR images of OMM2.3 mice before and after administration of ProCA32. No enhancement of liver metastases followed the administration of ProCA32. (**B**) T1 weighted and T2 weighted MR images of OCM1 mice before and after administration of ProCA32. No enhancement of liver metastases followed the administration of ProCA32. (**C**) M20-09-196 mouse before and after administration of Eovist. No enhancement of liver metastases followed the administration of ProCA32.


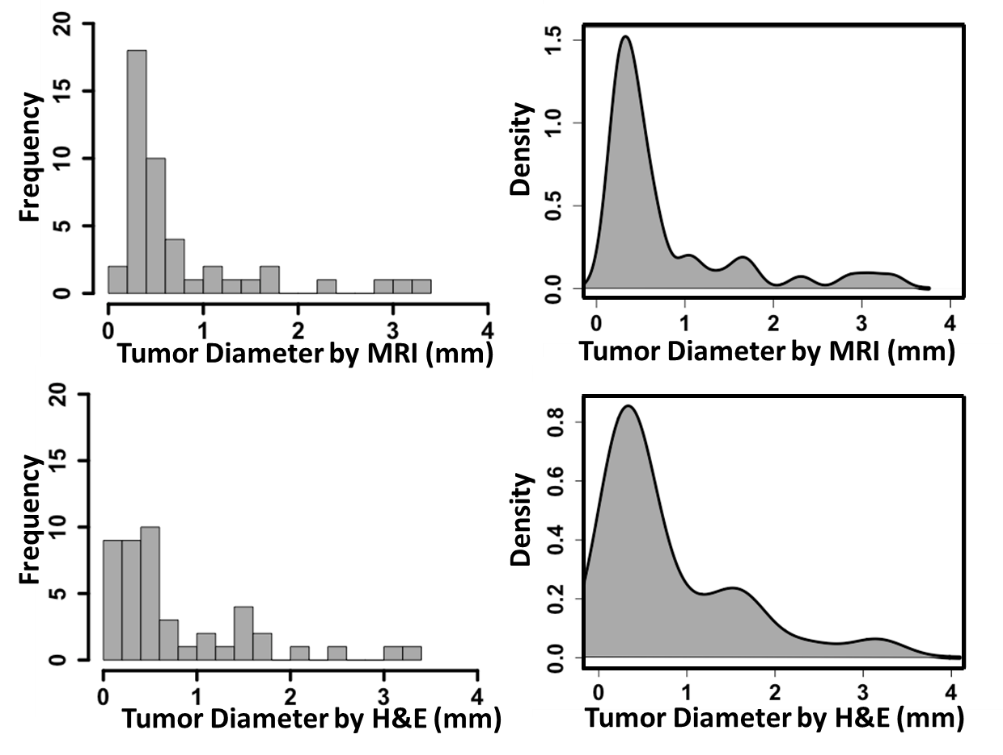


**Figure. S5**. **UM liver metastases size (tumor diameter) distribution**: histogram of size determined by MRI (top left), Kernel density plot of size determined by MRI (top right), histogram of size determined by H&E (bottom left), Kernel density plot of size determined by H&E (bottom right).
